# One health and human animal-bond intervention strategies- assessing veterinary-social service collaborations

**DOI:** 10.3389/fvets.2025.1656241

**Published:** 2026-01-07

**Authors:** Ronald J. Orchard, Elizabeth Scarbrough, Allison Crow, Matt Baldwin, Cassidy Moreau

**Affiliations:** 1 Department of Clinical Sciences, College of Veterinary Medicine, Kansas State University, Manhattan, KS, United States; 2 Topeka Chapter of the Street Dog Coalition, Topeka, KS, United States; 3 Veterinary Health Center, Kansas State University, Manhattan, KS, United States

**Keywords:** one health, human-animal bond, veterinary outreach, structural vulnerability, homelessness, interdisciplinary collaboration, trauma-informed care, community-based services

## Abstract

This study evaluates a novel interdisciplinary outreach model integrating veterinary care, social work, and public health services to serve unhoused populations and their companion animals in Topeka, Kansas. Grounded in structural vulnerability theory and One Health principles, the project examined the Street Dog Coalition’s (SDC) partnership with the Mobile Access Partnership (MAP), focusing on how the human-animal bond functions as a catalyst for trust, engagement, and care continuity. Using a qualitatively driven, mixed-methods design, the research team conducted 12 semi-structured interviews with beneficiaries, volunteers, program staff, and external collaborators, supplemented by descriptive service utilization data. Thematic analysis revealed six interrelated themes: the transformative power of the human-animal bond, trust-building as foundational to engagement, structural barriers to care continuity, tensions in the graduation process, emotional impact on providers, and the emergence of a relational ecosystem of care. Findings underscore the relational and structural dynamics of service delivery, highlighting the dual importance of compassionate, trauma-informed care and policy-level reforms. This research contributes to the growing evidence base for integrated One Health interventions and offers critical insight into how veterinary-social service collaborations can operationalize equity, dignity, and mutual healing in structurally vulnerable contexts.

## Introduction

In recent years, the field of veterinary medicine has begun to reckon with its role in broader public health systems, particularly in contexts of structural vulnerability. Access to veterinary care is increasingly understood not merely as a matter of service provision, but as a critical intersection of health equity, social justice, and public trust. Structural barriers—such as poverty, housing instability, lack of accessible transportation, and institutional policies that restrict access to both human and veterinary services for marginalized populations—continue to prevent unhoused individuals from obtaining care for themselves and their companion animals (1, 2). For unhoused individuals, whose pets often serve as vital emotional and psychological support systems, these access issues are particularly pronounced.

The 2018 Access to Veterinary Care Coalition report illuminated how deeply intertwined animal care is with human social determinants of health. Recent scholarship has further operationalized this connection, demonstrating how factors such as income, education, housing stability, and access to healthcare directly influence the well-being of both people and their companion animals (3). These findings reinforce the imperative to design outreach models that bridge veterinary and human services, particularly for those experiencing structural vulnerability. Building on this foundation, our study evaluates a unique, community-rooted outreach model that integrates veterinary care with social work and public health services. The program is a collaborative effort among the Kansas State University College of Veterinary Medicine, the Mobile Access Partnership (MAP), and the Street Dog Coalition (SDC), aimed at delivering holistic care to unhoused populations and their companion animals in Topeka, Kansas.

The Mobile Access Partnership (MAP) is a collaborative outreach initiative in Topeka, Kansas, that brings together multiple service providers to meet the immediate and long-term needs of unhoused individuals. Operating out of mobile units, MAP offers access to hygiene, housing navigation, healthcare, and social services in a low-barrier, relationship-centered setting. Within this ecosystem, the Street Dog Coalition (SDC) provides veterinary care for companion animals, using the human-animal bond as a bridge to holistic support. SDC not only delivers vaccinations, spay/neuter surgeries, and preventive care, but also fosters trust and engagement through compassionate, trauma-informed service. Together, MAP and SDC embody an interdisciplinary, place-based model that integrates veterinary, social, and public health responses to structural vulnerability.

This study is grounded in structural vulnerability theory (4), which posits that marginalization is not merely a function of individual behaviors or choices, but the product of systemic inequities embedded in institutions and policy. We also adopt a One Health approach that emphasizes the interconnectedness of human, animal, and environmental health (5). The integration of these frameworks enables us to ask not only what works, but also why it matters in the lives of those systematically excluded from care.

Specifically, this study explores how the human-animal bond can be leveraged to promote trust, engagement, and holistic well-being. It evaluates both the quantitative scope of services and the qualitative experiences of beneficiaries, volunteers, and providers, offering an evidence-informed model for interdisciplinary outreach. In this study, in step with other engagement scholars (6), we use the term beneficiary rather than client to emphasize the relational and care-based orientation of the Street Dog Coalition model. Unlike the transactional connotation of client, beneficiary reflects the program’s commitment to meeting people’s needs without expectation of payment or compliance, centering dignity and support rather than service consumption. Ultimately, we seek to understand: How does this community-based model address the barriers to care faced by unhoused people and their pets? And what are the implications for veterinary and social service integration moving forward?

## Method

### Design

This study employed a qualitatively driven mixed-methods design, grounded in structural vulnerability theory and One Health principles, to examine how a community-based veterinary outreach model serves unhoused individuals and their companion animals. The approach prioritized qualitative inquiry through thematic analysis, supported by descriptive quantitative data to contextualize program reach and participant demographics.

The overarching design reflected a critical relational paradigm, emphasizing power dynamics, care ethics, and equity in health access (7, 8). This allowed for both a systems-level evaluation of the outreach model and an exploration of how care is experienced and understood by stakeholders in real-world conditions of structural precarity.

### Program overview and context

The focal program, operated through a collaboration between the Street Dog Coalition (SDC) and the Mobile Access Partnership (MAP), provides integrated veterinary and human services in Topeka, Kansas. Kansas State University College of Veterinary Medicine contributes veterinary students, faculty, and infrastructure through its community outreach rotation. These clinics are hosted weekly via pop-up mobile events, typically co-located with human service providers such as housing navigators, harm reduction teams, and caseworkers.

The SDC-MAP model includes:

- Short-term services: preventive veterinary care (vaccination, deworming, microchipping), minor illness/injury care, pet food and supplies, and on-site case management for human needs.
- Long-term services: spay/neuter surgeries (facilitated via referral to brick-and-mortar clinics), continuity of care through follow-up events, and “graduation” into mainstream veterinary services for beneficiaries who have stabilized housing or income.
- Graduation model: Beneficiaries who demonstrate stability (e.g., secure housing and income, multiple repeat visits, pets fully vaccinated and altered) are encouraged to transition to traditional veterinary clinics. There is no fixed timeline—this transition is guided by beneficiaries readiness and supported through referrals and coaching.

Beneficiaries typically learn of the services via word-of-mouth, shelter case managers, or through MAP’s rotating event schedule. Intake is conducted on-site, consisting of brief documentation, consent, and verbal history gathering. All services are offered free of charge, intentionally removing financial barriers and reducing the structural stigma that may accompany the inability to pay for veterinary or human care. As of 2024, the program has served approximately 450 unique beneficiary households since its inception in 2020.

### Positionality and reflexivity

The research team included professionals from veterinary medicine, public health, and social work who were actively involved in program delivery and evaluation. This dual positionality necessitated ongoing reflexivity, supported through analytic memoing and journaling, to surface assumptions and mitigate power differentials in research relationships. The team was guided by an ethic of relational accountability (9), treating participants as knowledge-holders and collaborators (see Table 1).

**Table 1:** Description of interview participants by stakeholder group (*n* = 12) within the SDC-MAP model.

Category	Number of participants (*n* = 12)
Unhoused beneficiaries receiving veterinary and social services	7
Volunteer veterinary and social service providers	2
Program leadership and operational staff	2
External collaborators	1

### Sampling and participants

A purposeful sampling strategy (8) was used to ensure diversity across key program roles. We conducted 12 semi-structured interviews with participants from four stakeholder groups.

Table 2 description of study participants by stakeholder group. This table outlines the distribution of the 12 individuals who participated in semi-structured interviews, selected through purposeful sampling to ensure diverse perspectives. Participants represented four key stakeholder groups involved with the Street Dog Coalition model, including unhoused beneficiaries, volunteer providers, program leadership, and external collaborators (e.g., social service providers). Recruitment occurred through in-person engagement at SDC-MAP events, supplemented with follow-up communications. This stratified approach enabled a multi-perspective analysis of how services were experienced, enacted, and navigated across the ecosystem.

**Table 2:** Illustrative quotes supporting six themes derived from thematic analysis of stakeholder interviews in the SDC-MAP outreach model.

Theme	Definition	Representative quote
Transformative power of the human-animal bond	This theme captures how the emotional connection between clients and their pets functions as a life-sustaining relationship. Pets are viewed not just as companions, but as motivators, protectors, and emotional anchors. The bond often serves as the primary reason individuals engage with services and pursue stability.	“I want to get out of bed today because I have to take care of this animal… or I would have ended my life a long time ago if I did not have this animal depending on me.” (SDC.009)
Trust-building as a foundation for engagement	Trust within the SDC model is built through respectful, consistent, and nonjudgmental care. Clients frequently described being treated with dignity—often for the first time in institutional settings. This relational trust enables deeper engagement and contrasts with experiences of dehumanization in other systems.	“Recovery happens at the speed of trust. You cannot force someone to take the next step, but you can show up, every time, and prove you are not going anywhere.” (SDC.009)
Structural barriers to continuity of care	Despite successful engagement, systemic challenges—such as restrictive housing policies, lack of pet-friendly services, and transportation gaps—impede clients’ ability to maintain continuity of care. These barriers limit the impact of even the most compassionate service models.	“I had to choose between a bed in the shelter or staying with my dog. Of course, I stayed with my dog. But it means I stay outside, cold nights and all.” (SDC.003)
Tensions within the graduation process	The graduation model, while intended to signify progress, introduces emotional and logistical complexities. Clients often feel vulnerable leaving behind trusted relationships and face new obstacles in affording or accessing private veterinary care. The process reveals the fragility of post-program support systems.	“It’ll be kind of sad. They’ve been with me through so much. I know it’s a good thing, but I’m scared to do this without them.” (SDC.004)
Emotional impact on providers and volunteers	This theme reflects the emotional labor experienced by those delivering care. While stories of transformation and connection sustain commitment, providers and volunteers are also exposed to burnout, grief, and moral distress—requiring organizational structures for emotional support.	“The ones we cannot help are enough to make you want to quit.” (SDC.011)
Community and ecosystem of care	Beyond service delivery, SDC and MAP foster a relational community that offers safety, belonging, and mutual support. This ecosystem functions as a trauma-responsive environment where healing occurs through connection, and dignity is restored through consistent, relational care.	“Trauma created in relationships can only be healed in relationships.” (SDC.008)

### Volunteer roles and engagement

Volunteers, including veterinary students, practitioners, and lay personnel, are central to the outreach model. Their roles span from clinical duties (vaccination, exams, medical charting) to supportive services (intake, supply distribution, conversational engagement). Training in trauma-informed care, boundary setting, and ethical relationship-building is provided through orientation sessions, pre-event briefings, and mentorship by experienced staff. Frequency of volunteering varies—some individuals participate monthly, while others are embedded weekly across the outreach calendar.

### Data collection

Interviews were conducted between 2023 and 2024, lasting 30–60 min, and were recorded with participant consent. Interview guides explored:

- Experiences with veterinary and human services
- Role of companion animals in health and stability
- Trust-building and relational dynamics
- Barriers to access and feelings of empowerment
- Perspectives on “graduation,” continuity, and care transitions.

Interviews followed a semi-structured, responsive format (10), allowing flexibility while maintaining core prompts across stakeholder groups.

Quantitative data were extracted from SDC’s internal tracking database and included:

- Number and type of veterinary procedures performed
- Volume of beneficiaries served and pets seen
- Repeat engagement and follow-up visit rates
- Volunteer attendance and clinical involvement.

These metrics were used to contextualize qualitative findings—not as standalone analytic outputs, but to illustrate the breadth and structure of the service model.

### Data analysis

Interview transcripts were analyzed using reflexive thematic analysis (11), combining inductive coding with theoretical sensitization to structural vulnerability (4) and One Health (5). An initial codebook was collaboratively developed, then iteratively refined through team discussions. Themes and subthemes were constructed to preserve the narrative richness of stakeholder voices while identifying shared patterns across interviews.

**Figure 1:**
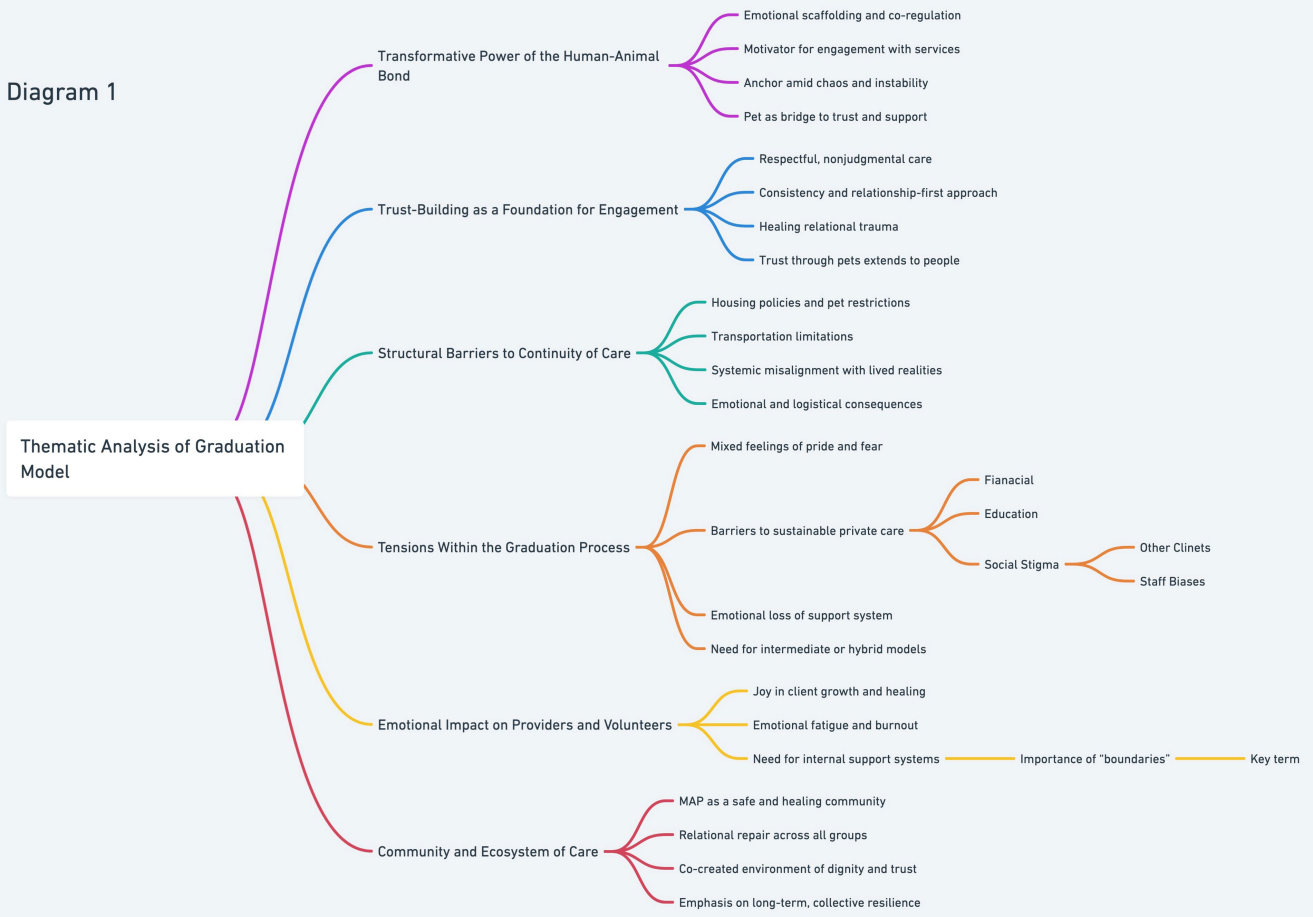
Illustrates six overarching themes that emerged from the qualitative analysis of the Street Dog Coalition community, spanning beneficiaries, volunteers, leadership, and service partners. These themes are supported by sub-themes drawn from participants’ experiences and narratives, emphasizing the central role of the human-animal bond. The figure highlights how trust, structural barriers, empowerment, emotional resilience, and community interplay to foster healing, stability, and engagement within a trauma-informed service model.

A visual summary of emergent themes is presented in Figure 1, accompanied by subthemes that illustrate the complexity of relational, structural, and affective dimensions of the outreach model.

To enhance trustworthiness and credibility, we employed:

- Thick description of service context
- Team-based coding and triangulation
- Limited member reflections
- Audit trails and analytic memoing
- Criteria of qualitative rigor outlined by Tracy (7), including sincerity, resonance, and coherence.

## Results

Findings from this qualitatively driven evaluation are organized into six overarching themes that reflect the complex ecosystem of relationships, care practices, and structural constraints within the Street Dog Coalition (SDC) and Mobile Access Partnership (MAP) model. Each theme emerged through inductive and deductive coding of interview transcripts, shaped by the study’s guiding frameworks of One Health and structural vulnerability. Themes are supported by subthemes and illustrative quotes, with descriptive program metrics providing contextual grounding.

Subthemes often intersected across major domains—for example, trust mediated by the human-animal bond appeared across multiple stakeholder groups. Rather than artificially separate these elements, we report them in their most salient thematic context while acknowledging their interrelatedness.

Table 2 illustrative quotes supporting six key themes derived from the qualitative analysis of beneficiary, volunteer, and provider experiences within the Street Dog Coalition model. This table highlights the core thematic findings, offering direct participant narratives that exemplify each theme. Quotes were selected to demonstrate the emotional, relational, and systemic dynamics shaping engagement, care continuity, and program outcomes. Together, these data illuminate the complex ecosystem of support fostered by the SDC and MAP initiatives.

### Theme 1: transformative power of the human-animal bond

Participants consistently described companion animals as stabilizing figures that provided emotional regulation, daily structure, and social identity. This bond often served as the entry point into human and veterinary services.

Subthemes:

- *Emotional anchors:* “She’s the reason I get up every day. When everything else falls apart, she’s still there.”
- *Mediators of trust:* “I would not let anyone help me. But I let them help my dog. And that opened the door.”
- *Caretaking as identity:* “I’m her person. That means something—even if I’ve lost everything else.”

### Theme 2: trust-building as a foundation for engagement

Trust was foundational for sustained engagement, particularly for individuals navigating past trauma and systemic exclusion. Participants valued consistent volunteers, nonjudgmental care, and relational continuity.

Subthemes:

- *Continuity of presence:* “I know they’ll be there every two weeks. Rain or shine. That matters.”
- *Nonjudgmental engagement:* “No one asked how I got here. They just treated me and my dog with respect.”
- *Predictability builds safety:* “I come back because I know what to expect. No surprises.”

### Theme 3: structural barriers to continuity of care

Participants described systemic barriers that shaped their access to both veterinary and human care. These included housing discrimination against pet owners, inflexible clinic policies, and lack of mid-tier veterinary options. Descriptive program data mirrored these challenges: Of the 451 pets served, 217 beneficiaries were lost to follow-up, and 43 declined services due to the mandatory spay/neuter policy. These figures reflect the fragility of sustained engagement when external systems fail to accommodate beneficiaries’ lived realities. Staff underscored the disconnect between their relational efforts and the broader policy landscape, stressing that relational care must be matched by structural reform.

Subthemes:

- *Housing-related exclusions:* “They said no pets. But I will not leave him. So I stayed outside.”
- *Binary care options:* “It’s either free here or $300 at the vet. There’s no middle ground.”
- *Policy dissonance:* “They told me I could not come back if my dog wasn’t fixed. But I do not have a car. How am I supposed to get that done?”

### Theme 4: tensions within the graduation process

The notion of beneficiary “graduation” to traditional veterinary care was emotionally complex. While the notion of graduation symbolized forward movement, it was often constrained by structural conditions beyond beneficiaries’ control. Among the 79 graduates, at least four returned due to financial or logistical difficulties—a pattern some providers referred to as “recidivism.” These experiences prompted critical reflection on the suitability of graduation as a programmatic goal:

“We need a middle step—something between outreach and full-on private care. A hand-off does not work if the other hand’s not there to catch them.” (SDC.009).

This tension illustrates that graduation, while well-intentioned, may prematurely withdraw services in contexts where beneficiaries remain structurally vulnerable. It challenges programs to rethink what “continuity of care” means when safety nets are limited, and when “success” depends not only on the readiness of the beneficiary but also on the responsiveness of downstream systems.

Subthemes:

- *Ambiguity of status:* “It’ll be kind of sad. They’ve been with me through so much. I know it’s a good thing, but I’m scared to do this without them.”
- *Loss of community:* “Being inside does not mean you do not still need help.”
- *Desire for transitional support:* “Just ‘cause I got a place to stay do not mean I can afford a $300 vet bill.”

### Theme 5: emotional impact on providers and volunteers

Volunteers reported experiences of fulfillment, moral distress, and relational labor that extended beyond technical veterinary tasks. They grappled with emotional boundaries and the ethical tensions of scarcity.

Subthemes:

- *Beyond clinical roles:* “I’m not just giving vaccines. I’m listening to someone’s life story.”
- *Emotional labor and limits:* “Sometimes I lie awake at night thinking about them. Did I do enough?”
- *Training gaps:* “We learn how to suture, not how to say ‘no’ to someone in crisis.”

### Theme 6: community and ecosystem of care

Participants—both beneficiaries and providers—framed outreach encounters as mutual acts of care and transformation. The clinic space became a site of belonging, healing, and shared humanity.

Subthemes:

- *Beneficiaries as change agents:* “She taught me how to be strong. I came to help, but I left changed.”
- *Volunteers as learners:* “Every clinic reshapes how I think about care.”
- *A temporary community:* “It’s not just a clinic. It’s a family for the day.”

## Discussion

This study sought to illuminate the complex interplay between structural vulnerability, the human-animal bond, and community-based service delivery within an outreach model serving unhoused individuals and their companion animals. Through a qualitative, stakeholder-engaged approach grounded in One Health and relational care frameworks, we identified key themes that reveal both the healing capacities and the limitations of current outreach paradigms. In the following sections, we situate these findings within relevant literature, evaluate the implications for practice and policy, and offer considerations for future research and programmatic design. Subsections are organized thematically to reflect and expand upon the six domains of analysis presented in the results.

### Human-animal bond as a therapeutic and engagement catalyst

The findings of this study strongly affirm the centrality of the human-animal bond (HAB) in shaping care-seeking behaviors, relational trust, and healing processes among unhoused individuals. Echoing established literature from One Health and human-animal interaction fields, participants consistently described companion animals as emotional anchors, sources of stability, and primary motivators for engaging with supportive services (1, 2). However, this bond extended beyond emotional comfort; it actively functioned as a relational conduit between beneficiaries and providers. In this context, pets became “co-beneficiaries,” shaping how outreach teams delivered care in ways that felt respectful, nonjudgmental, and dignified.

A prominent example is the intentional use of treats by volunteers—not merely as a clinical behavior management tool, but as a mechanism of relational alignment. This practice modeled what Hall (12) describes as relational attunement, a posture of care that centers presence, dignity, and mutual recognition. Hall’s concept, rooted in relational spirituality, is particularly salient in outreach models like the Mobile Access Partnership (MAP), which is organized in part by faith-based institutions, as well as Street Dog Coalition (SDC), where many volunteers and care providers share religious affiliations. In these contexts, relational attunement serves as a psychologically and theologically grounded framework for understanding how trust, reciprocity, and compassion facilitate healing.

For secular audiences, this approach is analogous to the well-established psychological theory of attachment (13), in which trust-building and security are fostered through consistent, compassionate, and responsive relationships. The HAB operates as both catalyst and medium for this process. Several participants described instances in which they initially sought help only for their pet but, through the process of engagement, found themselves accessing hygiene services, medical care, housing support, or mental health referrals.

Importantly, this study illustrates that the HAB is not simply a static or sentimental connection—it is an active force within the broader care ecosystem. The HAB helps establish psychological safety, lower social barriers, and invite layered engagement across domains of care. These findings align with cross-sectoral outreach literature that highlights animal welfare as an effective entry point for holistic service provision (4). However, this success is not automatic. The HAB must be intentionally activated through trauma-informed practice, culturally responsive care, and sustained, relationship-centered engagement.

### Trust as the currency of access

Trust emerged as the foundational condition for any form of engagement. Beneficiaries, many of whom described histories of institutional harm, viewed MAP-SDC events as rare spaces where trust could be rebuilt over time. This trust was not derived from organizational branding or eligibility screening processes, but from repeated human interactions—conversations, shared meals, and respectful treatment of their pets. In this sense, trust was relationally earned rather than institutionally bestowed.

This finding aligns with broader critiques of access-to-care models that emphasize intake processes, referrals, or checklists without accounting for the social–emotional labor required to bring structurally vulnerable populations into care (5). It also reinforces the need to understand care as an emergent process, not a transactional exchange. As one provider noted, “we are not just treating a pet; we are welcoming someone back into a system that’s failed them.”

### Structural constraints and policy disjunctures

Despite the strong relational foundations described by participants, several systemic barriers complicated the promise of continuity. One prominent tension was the mandatory spay/neuter policy required for continued access to veterinary care. While intended to advance animal welfare, this rule created a moral and logistical conflict for some beneficiaries, leading to disengagement. As previously noted, 43 beneficiaries declined services due to this policy, underscoring how well-intentioned rules may inadvertently replicate exclusion. This echoes literature in harm reduction and trauma-informed care, which warns against rigid policy enforcement that does not align with beneficiaries’ lived realities (6).

Program staff were acutely aware of this tension, often expressing frustration at being caught between relational commitments and policy mandates. Their narratives suggest a need for flexible, beneficiary-centered frameworks that honor both animal health goals and human complexity. Rather than viewing disengagement as beneficiary non-compliance, it should be interpreted as a signal that relational care must be matched by structural reform.

### Graduation as a transition, not a termination

Perhaps the most contested construct in this study was that of “graduation.” Originally conceived as an indicator of programmatic success, graduation denotes the moment when a beneficiary is referred out of outreach services and into traditional veterinary care. Yet both providers and beneficiaries challenged this framing. Several described it as abrupt, ill-fitting, or poorly timed given the complex realities of financial instability, housing precarity, and transportation barriers.

Among the 79 graduates, at least four returned due to financial or logistical difficulties—what some staff described as “recidivism.” Others expressed concern that the concept itself may be misaligned with a program grounded in structural vulnerability theory. As one participant stated, “we need a middle step—something between outreach and full-on private care. A hand-off does not work if the other hand’s not there to catch them.”

This suggests that graduation should be reimagined as a relational transition, not a service termination. A trauma-informed model might include follow-up check-ins, warm referrals with transportation support, and the opportunity for re-engagement without stigma. Such a model reflects best practices in housing and harm reduction sectors, where continuity and flexibility are central to sustained outcomes (7).

### Volunteers as relational agents and structural navigators

Volunteers played a pivotal role in mediating care relationships, modeling trauma-informed practices, and serving as boundary crossers between beneficiaries and systems. Their consistency, demeanor, and use of low-barrier language were frequently cited by beneficiaries as reasons they returned. Importantly, many volunteers took initiative to address gaps in care, such as printing pet health summaries for use at shelters or social service appointments.

Yet this relational labor is not without risk. Several providers expressed a need for more training around emotional boundaries, trauma exposure, and self-regulation. As one volunteer shared, “you want to do everything for them, but that’s not sustainable.” This echoes concerns in community health worker and peer navigator literature, where burnout and overextension are common due to poorly defined roles or lack of institutional support (8).

To ensure sustainability, programs like SDC should invest in robust training, clear scopes of practice, and support mechanisms for volunteers. This is particularly crucial given the increasing role volunteers play in navigating both veterinary and human service systems.

### Reframing programmatic success and future directions

Taken together, the findings invite a critical reexamination of how success is defined in community-based veterinary care. Traditional metrics such as graduation rates, number of procedures performed, or follow-up rates tell only part of the story. Equally important are metrics that reflect relational depth, trust-building, emotional safety, and responsiveness to structural vulnerability.

As such, future research should explore alternative indicators of programmatic impact, including longitudinal engagement, perceived trustworthiness of providers, and beneficiary-defined quality of care. Additionally, greater attention should be given to structural barriers—such as the scarcity of pet-inclusive housing and the lack of mid-tier veterinary services—which limit exit pathways for beneficiaries. Understanding these structural gaps will require cross-sectoral collaboration and research that bridges veterinary science, public health, and policy analysis.

Ultimately, the MAP-SDC partnership offers a promising model of relationally grounded, One Health-aligned outreach that centers both ends of the leash. But its success will depend on the degree to which relational insights are matched by institutional reforms and community-informed definitions of care success.

### Limitations and future directions

Several limitations merit mention. The study’s sample size was modest, and while purposively diverse, may not capture the full range of experiences. Interviews were limited to one geographic site, potentially limiting transferability. Nevertheless, the triangulation of perspectives across stakeholder groups and the integration of theory enhance the analytic rigor.

Future research might explore mid-range care models for beneficiaries transitioning out of outreach, examine pet-inclusive housing initiatives, or assess volunteer training protocols in multisectoral settings. Further attention to how structural reforms—not just relational adjustments—can create continuity across veterinary and human service systems is warranted.

## Data Availability

The raw data supporting the conclusions of this article will be made available by the authors, without undue reservation.
